# Sudden-onset headache due to reversible cerebral vasoconstriction syndrome

**DOI:** 10.1007/s10072-023-06863-0

**Published:** 2023-05-23

**Authors:** Jion Goto, Yoshifusa Abe, Shuichiro Watanabe

**Affiliations:** 1 Showa University School of Medicine, Tokyo, Japan; 2 Children’s Medical Center, Showa University Koto Toyosu Hospital, Tokyo, Japan; 3 Watanabe Children’s Clinic, Tokyo, Japan

**Keywords:** Headache, Adolescent, Reversible cerebral vasoconstriction syndrome

## Abstract

Reversible cerebral vasoconstriction syndrome (RCVS) is characterized by reversible segmental vasoconstriction of the cerebral arteries that spontaneously resolve within 3 months. Occurrence of RCVS peaks at around 40 years and the syndrome is common in women. Here, we report an adolescent boy case of RCVS.

A 13-year-old adolescent boy had sudden-onset and severe headaches during baseball activity 4 days before his hospital visit. He had recurrent thunderclap headaches and vomiting, which worsened when taking a bath or when sleeping at night. He had hyperuricemia, vitiligo vulgaris, and schizophrenia, which were treated with paliperidone and olanzapine. His physical and laboratory findings were normal. Brain magnetic resonance angiography (MRA) showed multiple and beaded-segmental vasoconstrictions in the bilateral middle cerebral artery (MCA) and right posterior cerebral artery (PCA), as shown in Fig. 1a. Contrast-enhanced computer tomography of the brain revealed some vasoconstrictions in the same MCA and PCA region (Fig. 1b). His headache was treated with oral amlodipine for 3 months. Headache severity and frequency were ameliorated. Multiple segmental vasoconstrictions on MRA improved without any symptoms five months after disease onset.

**Fig. 1:**
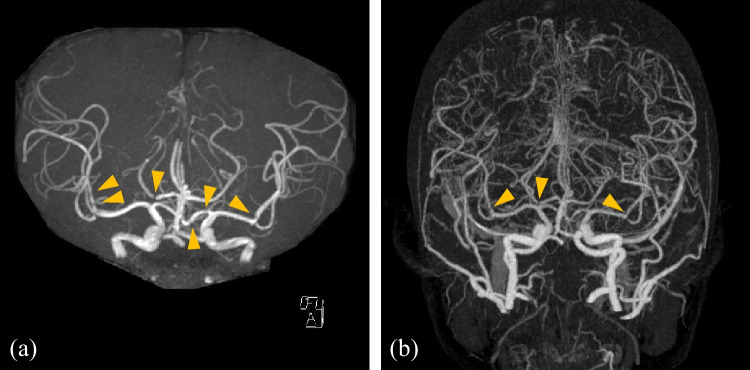
Findings of cerebral arteries. (**a**) Brain magnetic resonance angiography (MRA) findings. Brain MRA performed during hospital visit showed multiple and beaded-segmental vasoconstrictions in the cerebral arteries (arrow heads). (**b**) Brain contrast-enhanced computer tomography findings. Brain contrast-enhanced computer tomography performed one week after MRI showed multiple and beaded-segmental vasoconstrictions in the cerebral arteries (arrow heads)

Reversible cerebral vasoconstriction syndrome (RCVS) is characterized by reversible segmental vasoconstriction of the cerebral arteries that spontaneously resolve within 3 months [1], while headaches are usually described as the “worst ever” or thunderclap headache in patients with RCVS [2]. As our patient presented, bathing and exercise can trigger the headache in RCVS [1]. RCVS predominantly occurs in middle-aged females [1, 3], but approximately 85% of reported pediatric cases are interestingly adolescent boys [2]. This gender difference in RCVS remains uncertain. All patients need symptomatic management, such as eliminating any precipitating or aggravating factors [1]. Treatment with calcium antagonists relieves thunderclap headaches [1, 2]. Drugs for migraine treatment, such as triptans and ergots, can aggravate vasoconstriction [1]. Therefore, these drugs should be avoided to alleviate a thunderclap headache that is mistaken for a migraine attack. Other cerebral diseases, such as stroke and uncontrolled massive brain edema, lead to a poor prognosis [1, 2].

In conclusion, MRA must be performed if the pediatric patients have acute-onset and severe headaches, considering RCVS.

## Data Availability

Further anonymized data that support the findings of this case can be made available to qualified investigators upon request to the corresponding author.
